# Extra Supplement.—The Nursing Mirror

**Published:** 1888-11-24

**Authors:** 


### The Nursing Mirror.

All Contributions for this Supplement should be addressed "The Nursing Mirror," care of The Editor, 38, Old Jewry, E.C.

### En Passant.

THE GUILD OF ST. BARNABAS.—This Guild for Nurses has been steadily increasing in numbers lately, and has now secured a central office at 15, Brooke Street, Holborn. It is proposed to use the office as a sort of club-room, and every evening, from half-past six to half-past eight, tea and coffee will be provided at a reasonable charge, and medical books will be lent to members.

MENIAL WORK.—Please, we should like to know if nurses consider it menial work to drive an ambulance waggon? One of their number has performed this feat at Churchtown, and we hope she did not find it derogatory to her dignity as a nurse. The case arose thus: A boy had to be conveyed sixteen miles to the Workhouse Infirmary, and the driver was drunk. The nurse, with great common sense, took the reins herself, and did the best she could in an awkward position. Our own view of the matter is, that she is a woman to be admired.

THE ST. DENY'S SISTERHOOD.—There is a most interesting account of the Indian branch of this Sisterhood in last week's *Queen.* There has been considerable misunderstanding with regard to this Sisterhood and its nursing powers, which this concise little article quite clears up. The St. Deny's community was founded at Warminster in 1878. At the earnest request of Bishop French, of Lahore, two of the Sisters were sent to the Punjab to superintend a ladies' boarding school. In 1886 the Sisters were engaged in nursing typhoid, and feeling their lack of training they applied to England, when a trained Sister and Associate were immediately sent them. Sister Annie, who is superioress in India, undertakes the nursing of Lady Roberts's Home and the Officers' Hospital at Murree.

CHANGES AT CHARING CROSS.—Miss Gordon has got her work cut out for her to reorganise the nursing system at Charing Cross Hospital. The average number of patients in the hospital is 410, to nurse whom there is just now a staff of 19 sisters and 135 nurses and probationers. At present the probationers sleep at Norfolk House, but when the nursing is taken out of the hands of the St. John's Sisterhood in the spring, all the nursing staff will have to sleep at the hospital, where accommodation has yet to be erected for them. The St. John's Sisterhood—or rather the Norfok House branch of the All Saints' Sisterhood—have already commenced the nursing of the Metropolitan Hospital. While Charing Cross is building it ought to erect a convalescent home in the country, for the present plan of giving patients who are able to get about "permits" to take exercise in the Strand, is not conducive to order and respectability.

TRAINED OR NOT?—Dr. Bostock made a very grave and serious allegation against the Governors of the Chichester Infirmary at the last half-yearly meeting, and although the feeling of the meeting went against him, yet the doctor's statements were not replied to or proved unfounded. The Governors of the Infirmary advertise that they are prepared to send out to private families "efficient hospital-trained nurses," and Dr. Bostock says the nurses sent out are not hospital-trained nurses, and that, so far as he is concerned, he has not found them efficient, and, therefore, has had to send elsewhere when he has had to employ nurses for his patients. Three other medical men at the meeting expressed satisfaction with the nurses supplied. The Governors ought at once to prove to the public whether their course of training for nurses is efficient or not. We are able to confirm the justice of Dr. Bostock's allegations from our own experience.

THE NECESSITY FOR GENTLENESS.—The old days of awful cruelty to lunatics has surely passed away for ever, but there yet remains a touch of the old leaven in some of our asylums. Only last week an inquest was held at Birmingham, respecting the death of a late inmate of the Boro' Asylum. Deceased refused to attend morning service, and two nurses took hold of her arms and escorted her to chapel. A day or two later one of the arms was found swollen and inflamed; erysipelas supervened, and the patient gradually sank and succumbed to exhaustion. She was 75 years old and very feeble. A verdict of Accidental Death was returned; yet surely both those nurses must blame themselves.

NURSES AND PRESENTS.—The correspondence we have published lately on this subject has excited great attention and interest. It is generally felt that "Hand-clasp's" letter on page xxvii. of last week's issue exhausts the subject. Her view is that a true nurse prefers to work for love rather than for hire, and this, the public will be gratified to see, is the almost unanimous feeling of nurses in the present day. That being so, the public will be well advised to make themselves acquainted with the regulations of the National Pension Fund for Nurses, 38, Old Jewry, E.C. Every private patient who has the means must desire the opportunity of really helping every nurse who has rendered him signal service in the day of his necessity. This can best be done by placing a sum of money to the nurse's credit with the National Pension Fund for Nurses, as a patient will thus secure the maximum of benefit for the woman who has proved a ministering angel to him on his sick-bed. For this reason he would be less than human if he did not desire to secure his nurse equal comforts under similar trials. This can be best done in the way here suggested, and all grateful patients will do well to communicate with Mr. Edward Clifford, the honorary manager of the fund, at the address given above.

ANOTHER "VICTORIA HOME FOR NURSES," BOURNEMOUTH.—Last week Lady Glyn opened the Victoria Home for Nurses, Bournemouth, which is a branch of the Sanitary Aid Association, and has been established to provide a staff of efficient and well-trained nurses for cases in the town and district. Accommodation for thirty nurses, of whom about one half have already been engaged, is provided, and the promoters desire to secure a high class of nurses as members of their staff. The home has resulted from the proved usefulness of the system of district nursing which was established in Bournemouth a few years ago. A recent appeal produced the sum of £170 in cash, and £350 as a guarantee fund. Miss Dibben is the honorary secretary, Dr. D. C. Embleton is the chairman of the committee, and Miss Wingfield Digby and the Rev. Dr. Scott have rendered great service to the movement by permitting the matron and nurses to be boarded in the first instance at the Digby Institute. We would give a word of caution to the promoters of this excellent scheme upon one point. Instead of attempting to start an annuity and sick fund, as they propose, we would suggest that they should put themselves in communication with the council of the National Pension Fund for Nurses, 38, Old Jewry, E.C., and so save themselves from a serious pecuniary liability, the amount of which they evidently do not at present appreciate. Looking to the results of the late appeal, it is quite impossible to hope that anything like the sum requisite to establish such a fund upon a safe basis could be raised in Bournemouth. Affiliation with the National Pension Fund for Nurses would, however, solve the difficulty at once.

### Original Articles.

### ONE WAY OF NURSING.—Part I.

By an Australian Nurse.

The ripple of every movement, good or otherwise, for which the old country is responsible, finds its way, sooner or later, hitherwards.

We have our hospitals founded on English principles (with modifications and variations), our benevolent societies, our Girls' Friendly, even a Browning Society, and a young Charity Organisation ditto; but up to three years ago we had no Trained District Nursing. Besides being an imitative people, we are also one that loves a new sensation. Being so young, and with all our experience before us, it might reasonably be supposed that a new thing could not possibly raise in our incomplete minds the fury of zest it does in our battered, *blasé* old motherland, where everything has grown so stale, and the bloom seems to be rubbed off in the earliest babyhood of things.

Reasonable as might be such a supposition, it is nevertheless incorrect. We seize on a new experience here, and tear it to shreds with even more fervour and greed than one is wont to do at home; so that when the fact of the need of District Nursing—already patent enough to anyone who had any curiosity on the subject—was brought prominently forward by a leading divine and by a lady well known for her work among the slums in other spheres, it created quite a little sensation in fashionable circles.

We had not jaded ourselves by slum-hunting (that ripple had somehow failed to reach us), not found out for ourselves the dreary sameness of it, the dreary, overwhelming sameness of the sights, and sounds, and smells of it, that palls so soon; and which it requires a great deal more than excitement and the froth of philanthropy to overcome. We had not, however, yet proved this pastime and its worthlessness as such. So our new slum-sensation found almost a virgin soil to work in, and it did work, and to some purpose.

In a very short time a large and influential meeting was called at the house of a rich merchant here, and it certainly was a success, if numbers and zeal count for anything. A committee—which, if I remember aright, was to consist of twenty members—was formed on the spot, composed with hardly an exception of people of wealth and consideration, myself perhaps the only one who could claim neither the advantages of riches or position, could not so much as lay claim to the distinction of being an "old identity;" and I was only put on as having had some practical nursing experience in another continent.

However, I was put on, and then began for me a curious series of experiences and enlightenments. The hope of a small minority—myself among the number—was to found our institution on the lines of that of the Metropolitan and National Nursing Association, which were found to work capitally in London and other large cities at home; wherein only trained women of birth and education (under trained superintendence) are eligible for nurses.

Our poor here, even more than at home, require infinite tact, knowledge, and consideration in their treatment. They are independent (not to say bumptious) to a degree, and naturally all of a respectable bias; they therefore brook, even less than the poor in old lands, the interference of their betters, however much soever it may be for their good. And yet their condition is, if anything, more degraded than the old-world poor, being the outcome mostly of their own vices; not the result of generations of starvation and overcrowding—it is a condition that wants, if anything ever did, the raising, and guiding, and teaching, that only some constant, refining, enlightening influence could bring to bear on it. And we thought that this would, in some small measure, be supplied by the daily going to and fro among the people of gentle-born women, with gentle ways and voices and modes of thought; whose education has enabled them to learn their work as well as it is possible to learn it, and who would do their work for love of it, not merely as a means of livliehood; such women, in fact, with whom work would be working too, and who would have the capacity to impart their practical knowledge as well as to serve; which capacity will, as a rule, be found only among the educated.

We, this minority, also knowing from experience the snares and pitfalls that are likely to beset almost all philanthropic schemes—paramount amongst them being the radical danger of pauperising the poor—were anxious to guard against making our institution a nursing one pure and simple, with a *lending* store of all nursing appliances—blankets, linen, flannels, and attendance;—there being already, in our opinion, enough and to spare of relieving societies in the city, to all of which the nurses should be empowered to apply for aid for her patients.

*(To be continued.)*

### Jinny's Twin.

"No, ma'am, I aint got a father and mother; I aint got nobody but Monty, she's all the relation I've got."

The speaker was a tiny woman of five, all misshapen as to body and limbs, but angel-fair of face. Great dewy violet eyes lighted up the small oval countenance, and curling round the white brow were tendrils of chestnut hair—a human paradox of beauty and deformity.

They had brought little Jinny Nobody-knew-who to the Children's Hospital; she had been readily admitted, and all that modern medical skill could do—and that is no little—had been tried for the tiny sufferer, but in vain. Disease had clutched the poor limbs too tightly in its fangs for mortal man to unloose them. Jinny must die. Everybody knew it, and voices grew softer, eyes dimmer in the little presence which was fading so swiftly out of life.

Nothing was thought too good for the patient mite, and her cot was daily strewn over with offerings of flowers. Very often a single blossom—perhaps a pink—would be sent by one of the other little patients to "the little gal wot will never get no better." And Jinny's clever fingers would lay her flowers about in patterns on the coverlet, playing for hours at making gardens. That was before Monty came though.

"And who may Monty be?" inquired the visitor, who must have been a great personage, for everybody—the nurses, the sisters themselves, and even the doctors—stood stiffly at attention during her visit. She was sitting by Jinny's cot, and had been listening to the child's history told in her own simple fashion.

"Monty's my twin, ma'am!" was the answer; and Jinny's violet eyes sparkled, while her cheeks turned pink; then, with a musical chuckle, she went on: "Please, would you like to look at Monty? She's in here."

The coverlet was turned back, and there lay a very large wax doll, with eyes and chestnut hair ridiculously like Jinny's own, and at sight of which the visitor started.

"Sister Victoria says Monty's *very* like me," chattered Jinny, "and the funny doctor says we might be twins. And I suppose we be twins. I never had no sister, ma'am, but Monty's just as good as one. I do love her." Here Monty was feebly squeezed.

"Where did Monty come from?" asked the visitor, looking down at the two heads on the pillow.

"Oh! she's a lady's doll, Monty is. It was one day, just after I came here," said Jinny confidentially, "I was sitting up; somehow, I can't sit up now. I felt terribly bad, and a lady in a pink gown sat beside me; that was a nurse, you know. She told me stories about little children, then stories about Jesus, but my back and my legs wouldn't let me listen. Then some strangers came into the ward; a lady had brought her three little girls to see the sick children, and give us flowers. I forgot the pains in looking at the little ladies with their long fair hair, and their dancing feet, which would hardly let them walk softly through the ward. But when they came up to me they stood very still, and one whispered: 'Oh, this poor thing has been crying. She must be very ill.' Then they listened while the nurse told their mother how I had been brought to the hospital from old Aunty Jones' in the dreadful ugly back street. She told them, too, that Aunty Jones used to beat me, and wish I was dead, she did. And she whispered something low to the lady, but I don't think as it was about me, for the lady looked terrible sorry. When they heard I had nobody belonging to me, one of the little girls suddenly turned, and ran down the ward.

"'She has gone down to the carriage to fetch something for you,' said the others; 'perhaps the cake that we bought at Buszard's,' and they patted me and touched my hair. In a few minutes the other little girl came back; she ran right up, and put a beautiful doll into my arms.

"'Oh, Trixy,' said her sisters, 'you're not going to give her Monty, your own best doll!'

"'Yes,' said Trixy, 'I am. She shall have Monty to be a comfort to her; and, just see, they look like two sisters! Then she told me that when she went home she would send Monty's clothes, and she did. Monty has night-gowns, oh! and everything." Jenny, looking quite exhausted, ceased, and the visitor spoke kindly words to soothe the little sufferer, who presently dozed off sweetly, her head and Monty's, so pathetically alike, lying on the same pillow.

By-and-by, days and nights of cruel suffering came to little Jinny. Her small span of life was very near the end. At times, when there was a deceitful lull in the pains, Jinny would listen to the Sister's stories, especially to "that sweet story of old." And when the Sister told her gently that Jesus would want her soon to go home to Him, Jinny smiled fearlessly. It would be far better, she knew, than to go back to cruel Aunty Jones.

"But, Sister," Jinny's violet eyes were very wide, "when I go, I—shouldn't like to leave Monty behind. I couldn't do that. Will you promise to let her go with me?"

Sister Victoria was silent for a few moments; then her voice trembled as she spoke: "Yes, my child, I think I can promise you that. Your doll shall—go with you," and Jinny was satisfied.

"Would you like to see her, madam? She is looking as if in a sweet sleep." A door was pushed gently open, and the visitor who had sat by Jinny's cot many times since the first, followed the speaker into what seemed to be a little chapel. It was dimly lighted, but one could see, at the far end, a tiny altar on which was a cross. There were perfumes of exquisite flowers, and the eyes getting accustomed to the subdued light began to see a quantity of purest blossoms, fair white lilies, and wreaths.

"See! she lies here, poor little soul, her sufferings all over."

As if in peaceful slumber lay Jinny in the mortuary of the Children's Hospital. Her lovely eyes were hidden by the white lids, but over the fair face brooded a sweet content. Close to her—close as it used to be in life—lay Monty, the doll, with staring eyes. The visitor started, gazing in dumb surprise.

"It was her last prayer," whispered the Sister. "I could but grant it. She loved that doll with all the love pent up in her little lonely heart. They are to be buried together, she made me promise." And so they were, Jinny and her twin, Monty.

M. B. Manwell.

### Everybody's Opinion.

[*Correspondence on all subjects is invited. No communications can be entertained if the name and address of the correspondent is not given, or unless one side of the paper only be written on.*]

### NURSES AND PRESENTS.

T. A. writes: My opinion is that when patients really wish to make a nurse a present, they do so in spite of any rule of the institution against it; but it is generally the family from whom you receive the least consideration who are so sorry the rules of the home prevent them making the nurse a present, etc. I think it is very gratifying to receive a present other than money from a patient as some token of their appreciation; but nothing, I should think, can be more humiliating and insulting than to have money offered you. I knew a private nurse who had a very handsome watch presented her by a patient. Her matron heard of it, and said she must either return the watch to the donor or quit the institution. She was willing to take the latter alternative. However, as she was a good nurse, and always had excellent reports, she was allowed to keep the watch, and also retain her post at the institution. This shows how strict are such rules in some nursing homes. Whilst I think nurses should be allowed to receive presents from private patients, I consider no rule can be too severe, nor penalty too heavy, against a nurse accepting a present from a hospital patient; for I fear even at the present day there are some nurses who would allow the likelihood of a present to influence them in the amount of attention bestowed on a patient, and my experience goes to show me that unless the idea of making a present were encouraged it would soon cease to exist. As for a private nurse's salary being £20 per annum, that must be a mistake. I am sure no institution pays so little to a certified nurse. We can see advertisements week after week where the salary commences at £25. It is only the nurse who has not completed her training who does private work at such a low salary, and such nurses are a gross imposition on the public. Of course, an inexperienced nurse would not be sent to an operation case, but they are constantly sent to fever cases, who have never nursed it before. If we put it to ourselves, we should not like to pay two guineas a week for a nurse and then discover that she had neither practical nor theoretical knowledge of the case she had charge of.

"One of Miss Landale's Nurses" writes: Another nurse, "who was once at Bath," is anxious to say a few words on the subject that has been started in your paper. I worked under Miss Landale for rather more than a year, and never left a case without receiving some small present from my patient or her friends; these I immediately showed to our superintendent, who was always quite willing that I should receive such small gifts. Of coure, if money was offered, it was at once refused. The rule, I believe, ran thus—"No nurse to receive presents from patients or patients friends without permission of the lady superintendent." This permission, I can testify in my own case, was always most readily granted. Miss Landale certainly was, as your correspondent states, a "mother" to her nurses, and many a time have her kindly words of counsel come back to me since I left the home; and it was a real grief to me to leave so kind a friend. Miss Taylor is, I believe, in every way a worthy successor to Miss Landale, though of this I have had no personal experience.

Westmoreland writes: A fortnight ago I noticed with extreme surprise a paragraph in your valuable paper anent the Bath Nurses' Home. I think it would have been wise on the part of your correspondent (and our would-be friend), before penning such statements, to have at least ascertained their accuracy. As regards nurses and presents, although myself a nurse, I was not before cognizant of the fact that nurses cared or depended so much on any possible presents they might perchance receive from grateful patients. Certainly it is very pleasing to a nurse, when leaving a convalescent patient, whom, under the doctor's instructions, she may have successfully nursed through a critical illness, to receive some small token of gratitude as a memento; but, as a general rule, I am positive that nurses neither look for nor expect anything more than to see their patients recover health and strength, and in watching day by day with interest the progress made towards recovery, a nurse feels amply repaid for any sleep or recreation she may have lost during the critical period. Probationer nurses receive at the Home £20 a year, rising after the first year, and I believe that in a majority of the best nursing institutions the salary for a first-year probationer trained by a Home is the same. Your correspondent then proceeds to state that after an illness of a month or six weeks in the Home, the nurses, if not better, are sent to the hospital. From experience I can state that is strictly correct, but the nurse in question went to the hospital because the doctor who so kindly gives his services to the nurses strongly advised it, for treatment the nurse could not have obtained in the Home before undertaking a long journey home; and further, it was with the entire consent of the nurse's friends. No other nurse, to my knowledge, was ever sent from the Bath Nurses' Home to a hospital, and the nurse who was sent to the Bath Royal United Hospital will ever be grateful to the managements of both valuable institutions for the benefits received whilst with them. You ask for the opinions of nurses on this vexed question. My own opinion is that it would be advisable for outsiders to leave matters alone which they clearly do not properly understand, but leave committees and nurses to manage their own affairs. I fully sympathise with our unknown friend's undoubted good intentions. In future, I would recommend any would-be friends to at least consult the nurses' feelings on matters concerning only themselves before endeavouring to benefit them. I take it for granted that I am the nurse referred to in the article as being sent to the hospital, and can honestly state that I feel nothing but gratitude for the great kindness and consideration shown to me in both Home and hospital.

M. E. F. asks :— Would A. E. prefer a rule to the effect "That no one need apply for a nurse unless they can guarantee a present, expensive or otherwise, for the nurse, in addition to the fees for the institution ; as we cannot afford to give them more than £20 a-year, and as a body they are not thrifty enough to put by for a rainy day ?" It is a pity that a nurse is not quite conscientious enough to adhere to the rules of the institution she has joined of her own free will. (I most sincerely hope our Lady Superintendent will never exact the observance of such a rule from us. At present we are at liberty to do as we choose, our dear matron trusting to our honour not to abuse the privilege. I may here observe for the credit of the institution, that I have never known it to have been abused.) Should such a calamity happen as the necessity for the enforcement of such a rule, I would be one of the first to advocate it. Handclasp's remarks are so very much to the point, so aptly describe my opinions on the subject, and that I hope, of the majority of our fellow nurses, that I need not enlarge upon them at all, and would advise all those nurses who are in favour of present-giving to read them and give them their earnest consideration.

A Stoke Nurse writes :—In answer to Handclasp's letter in your last week's issue, I, as a nurse of several years' experience in private nursing, can safely say I have never met with the class of nurses referred to by your correspondent. It appears she is not against receiving presents, providing they are not called by that name, viz., by remaining at her patient's house for a week at his or her expense, when her services are not required, or as she suggests being sent to her own home, carriage paid. Where would your correspondent draw the line ? If that is not in the category of presents I cannot define it. Truly no woman is obliged to be a nurse, but having joined the profession, I think, as a rule they are too well educated not to know the duties to their patients, and will do all in their power to uphold the dignity of the institution to which they belong. I cannot think that nurses receive presents as a matter of business, but as an appreciation for services rendered, perhaps in some cases in excess of what they are required to do by the rules of the institution.

### OUR NURSE.

By a Patient at St. Thomas's Home.

Who is it, with a smile so sweet,
Will gently raise the under sheet,
And place hot bottles 'gainst your feet,
Our nurse.

Who, with clean dress and apron white,
Approaches with a step so light,
She never wakes you up at night,
Our nurse.

Who is it, with such peaceful ease,
Measures the liquid (three degrees),
And says, "Now take your medicine, please,"
Our nurse.

Who with thermometer enjoys
To "take the blood" of all the boys,
And warns us "not to make a noise."
Our nurse.

Who, when the pain and anguish rack
Our feeble and rheumatic back,
With friction soothes each sharp attack.
Our nurse.

When in the night we sleeplesss lie,
And scarce can close the weary eye,
Who, gently, morphia can apply,
Our nurse.

Who brings our meal of dainty (?) food,
And soothing each unquiet mood,
Whispers, "Now, this will do you good,"
Our nurse.

Who never from her duty shrinks,
And at our dissipation winks,
Allowing *microscopic drinks,
Our nurse.

Who dresses all our wounds and sores,
And balm of consolation pours,
To ease the pain which one endures,
Our nurse.

Who is it lifts your weary head,
And smooths the bread-crumbs from your bed,
And crumbs of comfort gives instead,
Our nurse.

Who is it, when you have to part,
And on your homeward journey start,
You will enshrine in grateful heart,
Our nurse.

J. S. B.

* Our allowance of whisky was 2 oz. per diem.

### Appointment.

[It is requested that successful candidates will send a copy of their applications and testimonials, with date of election, to The Editor, The Lodge, Porchester Square, W.]

General Hospital, Barbados.—Miss Margaret Haynes, who has completed her training at the Royal Free Hospital, Gray's Inn Road, W.C., has been appointed matron of the General Hospital, Barbados. The hospital contains 200 beds, and is nursed by black and coloured nurses. We congratulate Miss Haynes and also the lady superintendent of the Royal Free Hospital upon this selection for so important an office, and we wish Miss Haynes much happiness and success.

### Presentation.

On November 15th the medical staff of the London Hospital presented two handsome dressing-bags to Miss M. Burke and Miss Thackwaite, on the occasion of their departure for Australia. The bags were fitted with silver and crystal, and all articles bore the monogram of the owners. Both Miss Burke and Miss Thackwaite trained at the London Hospital, and have since acted there as sisters. Their departure is regretted by many friends.

### Lectures.

On November 28th and 30th, and December 3rd, 5th, and 7th, Mr. Victor Horsley will lecture at the London University, at five p.m., on "Epilepsy." These are the Brown Institution lectures, and are free to the public. It is to be hoped there will be a better attendance than there was last year, for Mr. Horsley is a splendid lecturer, and can do justice to his subject.

### Vacancies.

The following vacancies are announced :—

**Matron.**

Buckinghamshire General Infirmary ; £60.

**Lady Superintendent.**

Nurses' Institute, Canterbury.

**Nurses.**

Cardiff Infirmary.

Home Hospital, Eastbourne.

Nurses' Home, Beckenham.

Birkdale Home, Southport.

Miss Boor's Home, Nottingham.

Kingston Home, Hull.

Nottingham Nursing Institution.

Brecon Infirmary ; £18.

St. Pancras Workhouse ; £20.

Cheltenham Nursing Institution.

Royal Surrey County Hospital, Guildford (assistant) ; £12.

Hospital for Epilepsy and Paralysis, Regent's Park ; £20

Hollond Institution, Nice.

Rotherham Hospital and Dispensary (assistant); £18.

Croydon General Hospital ; two.

Guildford County Hospital ; £20.

St. Bede's House, Fairfax-road.

Canterbury Nurses' Institute.

Nursing Institution, 96, 97, 98a, Wimpole Street, London, W., Mr. Wilson has several vacancies for thoroughly-trained Nurses.

Croydon Hospital ; two.

Canterbury Hospital ; £25.

Children's Hospital, Kingsholm, Gloucester ; £21.

General Hospital, Tunbridge Wells ; two ; £25 and £22.

Victoria Home for Nurses, Bournemouth ; several : £25.

Wrexham Union ; £25.

Home for Epileptics, Maghull, near Liverpool.

**District Nurse.**

Leeds.

**Probationers.**

Blackheath and Charlton Cottage Hospital.

Bootle Borough Hospital.

### Notes and Queries.

To Correspondents.—1. Questions may be written on post-cards. 2. Advertisements in disguise are inadmissible. 3. In answering a query please quote the number. 4. If a private answer is desired, a stamped addressed envelope must be forwarded.

**Queries.**

(85) Can any of your readers tell me of an occupation suitable for a woman with one arm. Remuneration not necessary.—*Doris.*

(86) Nurse Nelson writes : Could you tell me where or how I could get all particulars abour nurses going out to the Egyptian Hospital, and are any nurses wanted there or at the Cape Hospital just now ? Could you find out how much they are paid per month, and what extras are allowed ?

**Answers.**

(80) *Childhood's Ailments.*—"Mater" had better get Goodhart's "The Diseases of Children." Price, 10s. 6d. Churchill.

(81) *Hospital Training.*—Women are taken at nearly every large hospital if they pay a fee. M. Britton had better settle which hospital she would like to enter, and apply there.

(82) *Surgical Training.*—King's, The London, and other hospitals would take a private nurse, but not for such a short period as six weeks.

(84) *Book on Midwifery.*—We should advise Doris to get Barnes' "A Manual of Midwifery," with illustrations. Price 6s. Smith and Elder.

